# Hydroxyurea—The Good, the Bad and the Ugly

**DOI:** 10.3390/genes12071096

**Published:** 2021-07-19

**Authors:** Marcelina W. Musiałek, Dorota Rybaczek

**Affiliations:** Department of Cytophysiology, Institute of Experimental Biology, Faculty of Biology and Environmental Protection, University of Lodz, Pomorska 141/143, 90-236 Lodz, Poland; marcelina.musialek@edu.uni.lodz.pl

**Keywords:** hydroxyurea, replication stress, ribonucleotide reductase, DNA replication checkpoint, cell cycle arrest

## Abstract

Hydroxyurea (HU) is mostly referred to as an inhibitor of ribonucleotide reductase (RNR) and as the agent that is commonly used to arrest cells in the S-phase of the cycle by inducing replication stress. It is a well-known and widely used drug, one which has proved to be effective in treating chronic myeloproliferative disorders and which is considered a staple agent in sickle anemia therapy and—recently—a promising factor in preventing cognitive decline in Alzheimer’s disease. The reversibility of HU-induced replication inhibition also makes it a common laboratory ingredient used to synchronize cell cycles. On the other hand, prolonged treatment or higher dosage of hydroxyurea causes cell death due to accumulation of DNA damage and oxidative stress. Hydroxyurea treatments are also still far from perfect and it has been suggested that it facilitates skin cancer progression. Also, recent studies have shown that hydroxyurea may affect a larger number of enzymes due to its less specific interaction mechanism, which may contribute to further as-yet unspecified factors affecting cell response. In this review, we examine the actual state of knowledge about hydroxyurea and the mechanisms behind its cytotoxic effects. The practical applications of the recent findings may prove to enhance the already existing use of the drug in new and promising ways.

## 1. Introduction

Hydroxyurea (HU) is a well-known genotoxic agent whose biological impact on living organisms has been known for almost a century [1]. Its anti-tumor abilities were reported for the first time as early as the 1960s [2,3]. Since then it has become a well-established drug used to treat a variety of diseases, such as in combination therapy for brain tumors [4], myeloproliferative disorders [5], and sickle cell anemia [6,7,8], and lately it was shown to improve spatial memory in an Alzheimer disease (AD) mouse model, which makes it a promising treatment for delaying cognitive decline in AD [9].

Technically, HU is an inhibitor of DNA replication [10]. It affects the activity of ribonucleotide reductase (RNR) by disrupting the proton-coupled electron transfer that catalyzes the production of new deoxyribonucleotides (dNTPs). In this process, HU activity (and the consequences thereof) is most prominent in the S-phase of the cell cycle, where it prevents the synthesis of daughter strands of DNA and, as a result, causes cell cycle arrest and checkpoint activation [11,12,13,14,15]. Due to the fact that these events are easily reversible, HU is also commonly used in scientific research for the synchronization of cell lines. Higher concentrations or a longer incubation time can cause a variety of malfunctions, such as accumulation of DNA damage sites (which later result in chromosome damage) or generation of reactive oxygen species (ROS), which induces cytokinesis arrest by oxidative stress [16,17] and contributes to the genotoxicity of HU.

The presence of hydroxyurea in cells primarily activates the S-phase checkpoint, which delays mitosis and arrests the cell cycle progress until DNA replication is finished and any occurring DNA damage is fixed. This checkpoint activation is crucial for proper cell proliferation and it is carefully regulated by signaling kinases, e.g., ATR in higher eukaryotes, Mec1 in *Saccharomyces cerevisiae*, or Rad3 in *Schizosaccharomyces pombe*. The exact ways in which the kinases recognize specific DNA lesions or damage sites are still unknown and are widely studied. The pathway, however, is highly conserved within eukaryotes [18,19]. Recently, the ataxia telangiectasia and Rad3-related (ATR) kinases have been shown to also play a vital role in the stabilization of stalled replication forks [20,21,22]. The absence of replication checkpoint leads to a vast range of malfunctions, with mitotic catastrophe being one of the most vague [23,24], as it may lead to cancer development or cell death. Cells that are checkpoint mutants are usually very sensitive to hydroxyurea, while those with the S-phase checkpoint intact tend to be mostly unaffected by HU once the drug is removed.

Many inhibitors of replication, such as HU, are in fact used as active agents in chemotherapy [4,25,26], so there is a very practical value in the theoretical knowledge of the exact mechanisms by which they affect living cells [27,28]. The cytotoxic effects of HU are usually linked to the accumulation of DNA strand breaks [29,30] or to the reactive forms of HU by-products after prolonged incubation, such as ROS [16,31,32,33]. In this review, we summarize recent findings regarding the impact of hydroxyurea on living organisms. The mechanisms that, on the one hand, are able to effectively cause stress and kill the cell may—on the other hand—also be leveraged to the advantage of cancer therapy.

## 2. The Mechanism of RNR Inhibition by Hydroxyurea

**Figure 1:**
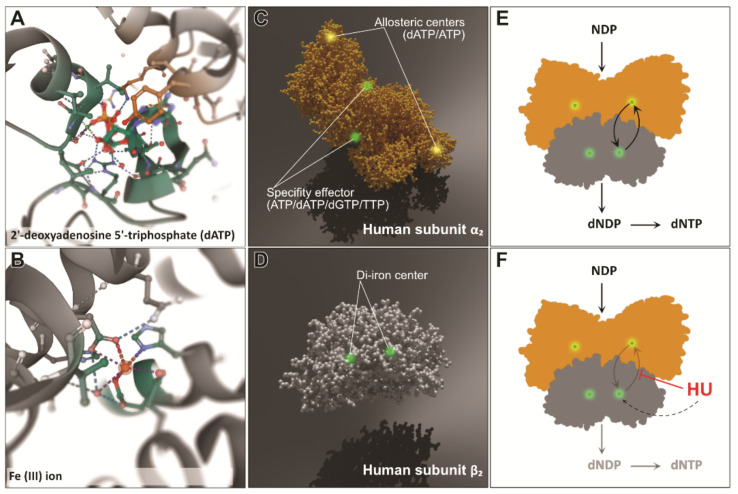
The structures of two homodimeric subunits of ribonucleotide reductase and the impact of hydroxyurea on the PCET transfer: (**A**) the dATP in the allosteric center of subunit α2 of human RNR (PDB: 2WGH) [36]; (**B**) the Fe(3+) ion center of the human β2 subunit (PDB: 2UW2); (**C**) a 3D model of the α2 subunit with the allosteric sites and active (substrate) sites (PDB: 2WGH); (**D**) a 3D model of the β2 subunit with the di-iron center placement marked (PDB: 2UW2). (**E**) Bidirectional PCET can occur only when both subunits form an α2β2 heterodimeric structure. This binding allows for substrate–effector interaction. (**F**) Hydroxyurea inhibits PCET within RNR by proton-coupled electron transfer, most probably mediated by a hydrogen-bonded proton wire [35]. The images in (**A**,**B**) were generated using Mol*Viewer [46].

DNA synthesis and repair depend heavily on the activity of ribonucleotide reductases (RNRs)—enzymes that utilize proton-coupled electron transfer (PCET) reactions to catalyze the reduction of ribonucleotides to deoxyribonucleotides [34,35]. The reaction catalyzed by RNR is an irreversible and crucial step in the process of building the free nucleotide pool—the substrates needed for DNA replication as well as repair. Class Ia RNRs (present in prokaryotes as well as in eukaryotes) consist of two homodimeric subunits that cooperate in order to perform the catalysis. Their crystal structures have been shown to be similar between species such as *Escherichia coli*, yeast, mice, and humans [36]. The smaller subunit, a homodimer called β2, holds a stable diferric-tyrosyl radical cofactor (generated by the oxidation of a di-iron cofactor (Figure 1B,D)). The larger subunit, also a homodimer called α2 (Figure 1A), contains an active (catalytic) site as well as two allosteric regulatory sites [37,38]. The simplest instance of an active form of RNR, as found, for example, in *E. coli*, is set up as an α2β2 heterodimer (Figure 1E). This configuration is the basis of RNR activity in pretty much every living organism [37,39,40]. The α2β2 complex allows for bi-directional long-distance radical transfer (RT) to occur [41,42]. Radical transfer from subunit β2 to α2 generates a thial radical in subunit α2 and catalyzes the reduction of NDP to dNDP. The reverse transfer (from α2 to β2) regenerates the tyrosyl radical in the β2 site [41,42,43,44,45].

The regulation of RNR activity depends on the activity of allosteric, transcriptional, and post-translational factors that together maintain the proper level of free dNTPs within the cell. At the allosteric level, dATP binding to the allosteric site at the N-terminus of α2 (Figure 1C) inhibits overall RNR activity by changing the binding patterns of subunits α2 and β2, while ATP binding does the opposite and restores RNR activity [37,44,47]. This feature helps in the regulation of the pools of free dNTPs and prevents the overstocking of the cell [48,49,50,51]. The auto-regulatory mechanism is based on the dNTPs giving ″feedback″ to alter RNR preferences and produce a specific deoxyribonucleotide, thus maintaining the proper balance between the levels of dATP, dGTP, dTTP, and dCTP [37].

Ribonucleotide reductase can be also inactivated by chemical factors, such as hydroxyurea, hydroxylamine, or N-methylohydoxylamine—molecules that are known to be radical scavengers. Hydroxyurea has been reported to reduce the diferric-tyrosyl radical center within the β2 subunit [52]. The reaction mainly proceeds by electron transfer from HU to RNR [16]. Hydroxyurea has been shown to be active in the majority of living organisms, mostly because the free radical mechanism of reaction catalysis in RNR is conserved between prokaryotic and eukaryotic cells. The mechanism by which HU reduces the radical in RNR is poorly understood most of the time [53]. Radical sites are quite well hidden within the body of subunit β2 [41,42,44], which contributes to their longevity [25]. Hydroxyurea has been suggested to be able to penetrate the protein (as a fairly small molecule) and reach the radical site. Another theory suggested long-range electron transfer, given the fact that much larger molecules (e.g., resveratrol) are able to inhibit RNR in the same way as HU [54,55,56]. Recently, kinetic analysis and reaction-induced FT-IR (RIFT-IR) spectroscopy have revealed new evidence of the mechanism. It has been proposed that the reaction involving proton transfer is facilitated by an extensive proton wire composed of a hydrogen-bonded network that includes aspartate and glutamates. The reaction in which a proton and electron are donated to a radical site in the RNR subunit β2 seems to be more direct and independent of the proton wire [35].

The mechanism of RNR inactivation by HU as the main cause of HU-induced replication stress is widely accepted; however, recent studies suggest that there may be more to the picture than meets the eye. Though some research studies indeed showed that HU reduces the concentration of dNTPs [57], others found that dNTP levels were mostly unaffected, and yet—at the same time—the inhibition of transcription or translation was observed [10,58]. Also, the inactivation of RNR may also stem from a more general ability of HU to alter enzymes that use metals as catalytic agents. HU has been reported to alter Fe-S centers of several cellular enzymes, many of which maintain a normal cell metabolism, so the inhibition of replication may be the effect of more “distant” pathway alteration. This seems to be supported by the fact that relatively low HU concentrations (which do not effectively inhibit RNR) still induce an S-phase checkpoint activation. Another way hydroxyurea may be toxic to the cell is its decreasing stability in water. Over time or in the presence of heat, HU breaks down to N-hydroxyurethane, hydrogen cyanide, nitric oxide, and/or peroxide [5,59,60,61]. It has also been proposed that HU may indirectly induce other reactive oxygen species (ROS) via interactions with Fe or other metals [16].

## 3. Stalled Fork Maintenance, Cell Cycle Checkpoint Activation, and Production of New dNTPs in Response to HU

**Figure 2:**
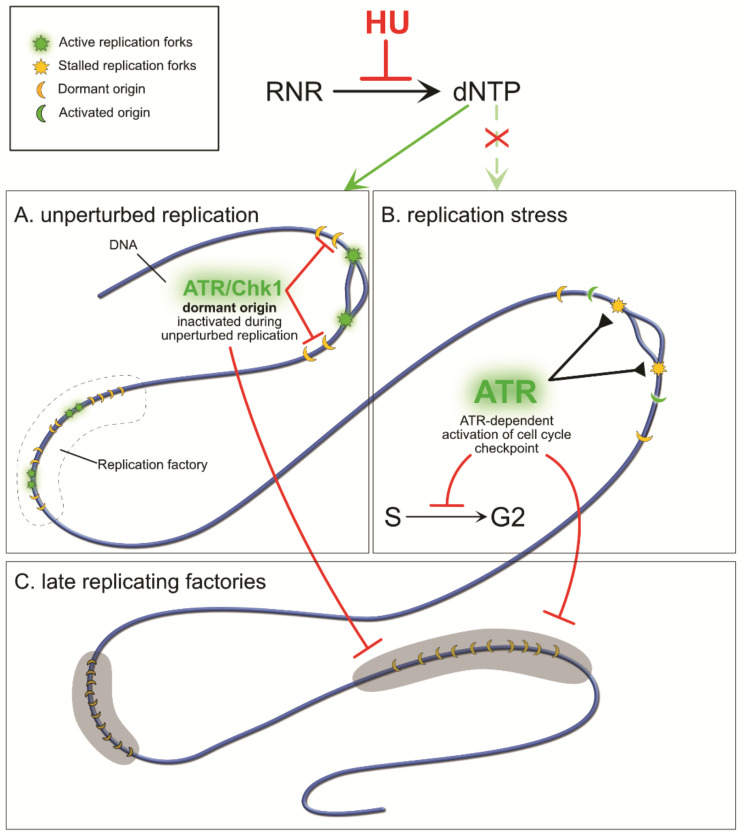
The cell response to HU-induced depletion of dNTPs during replication. Origins licensed on the DNA strand are grouped into replication “factories” the activation of which is strictly defined in time, from early- to late-replicating. In normal conditions, only a small percentage of origins will become activated and start to replicate DNA; the other origins remain dormant and will be replicated passively unless they are needed (**A**). If the currently replicating factory already has the desired amount of active forks, an ATR/Chk1 signaling mechanism limits dormant origin firing and also limits the activation of other factories that are supposed to replicate later (**C**). In the absence of free dNTPs (**B**), active forks stall and expose fragile ssDNA (**B**,**C**). ATR kinases stabilize stalled forks and also trigger the signaling pathway of the S-phase checkpoint, which arrests the cell cycle, inhibits origin firing in inactive factories, and restores RNR activity (**C**). The cell is unable to enter mitosis unless DNA replication is finished and any damage repaired (**C**).

**Figure 3:**
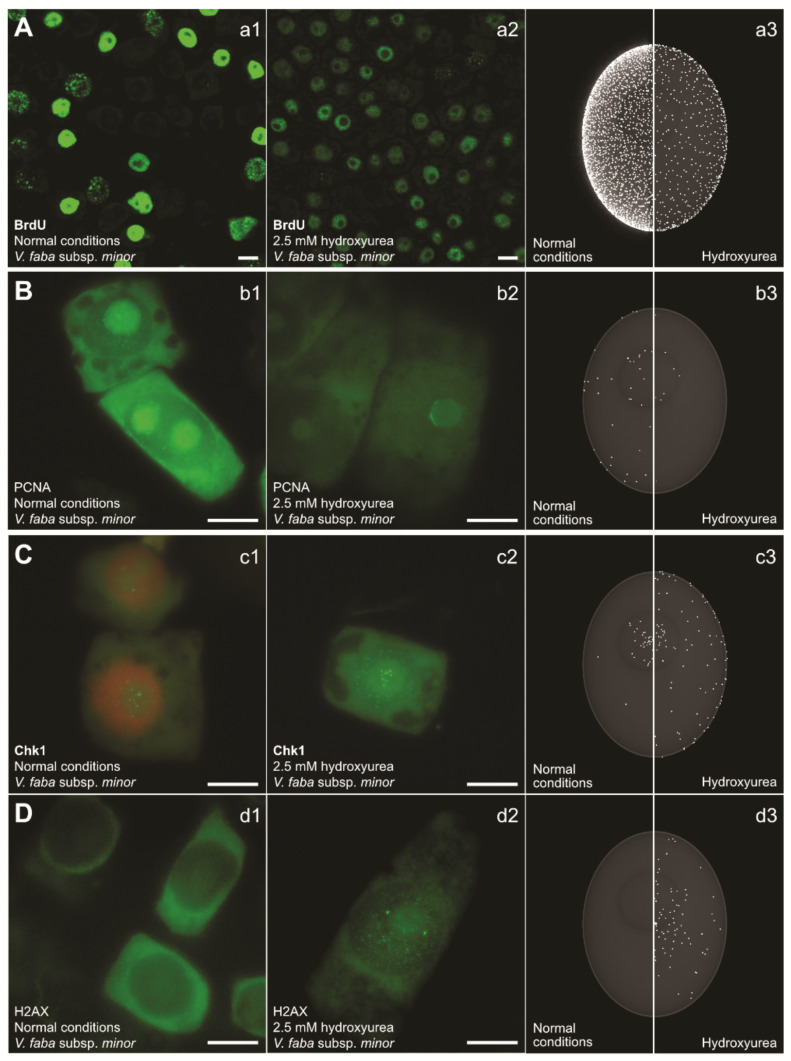
HU changes the activity of replication as well as several key proteins in root meristem cells of *Vicia faba*. (**A**) Nuclear DNA labeled with 5-bromo-2′-deoxyuridine (BrdU; **a1**–**a3**); (**B**) PCNA (**b1**–**b3**); (**C**) Chk1 kinase (**c1**–**c3**). The DNA within the nucleus is detected with propidium iodide (orange; **c1**). The control levels of Chk1 are consistent with reports that cells maintain a basic level of kinases in order to prevent dormant origin firing (**c1**,**c3**). (**D**) Histone H2AX (**d1**–**d3**), which is a hallmark of double-strand DNA damage (H2AXS139ph), shows increased levels after HU exposure (**d2**,**d3**). The presented factors were detected using immunocytochemical staining after 32 h incubation in water (first column, normal conditions; **a1**,**b1**,**c1**,**d1**) or in 2.5 mM HU (second column; **a2**,**b2**,**c2**,**d2**). The third column contains 3D renderings of BrdU/protein localization within the nucleus, based on statistical analysis of experimental data and microscope observations (**a3**,**b3**,**c3**,**d3**). The left side of the column shows the agent’s activity under normal conditions and the right panel shows the activity after HU treatment. The experimental procedures for immunocytochemical detection of: (i) PCNA, (ii) Chk1 (phosphorylated on serine 317), and (iii) H2AX (phosphorylated on serine 139) were identical (BrdU detection required hydrolysis with HCl). The experimental procedure for the DNA replication assay with BrdU (on entire cells) was as follows: control and HU-treated (2.5 mM, 24 h) seedlings were pulsed for 30 min with 30 µM BrdU solution at 20 °C in the dark. Excised 3 mm long meristems were then washed with ice-cold Tris buffer (10 mM Tris, 10 mM EDTA-2Na, 100 mM NaCl, pH 7.2) for 5 min and fixed for 45 min at 4 °C in freshly prepared 4% paraformaldehyde. After fixation, root tips were washed, squashed onto slides, and treated with 1.5 M HCl (for 1.5 h at 20 °C, for partial denaturation of nuclear DNA). In contrast, detection of PCNA/Chk1S317ph/H2AXS139ph required an enzymatic maceration step, i.e., incubation in a citric acid-buffered digestion solution for 45 min at 37 °C. Subsequently, root tips were washed again, squashed onto slides, and treated with the following primary antibodies: (i) mouse monoclonal anti-BrdU (Sigma-Aldrich, Saint Quentin, France) diluted in Tris-buffer (1:50) or (ii) the rabbit monoclonal antibodies anti-PCNA (Abcam, Cambridge, United Kingdom), anti-Chk1(S317ph) (Cell Signaling Technology, Beverly, MA, USA), and anti-H2AX(S139ph) (Cell Signaling Technology; Beverly, MA, USA; 1:250), diluted in PBS. Following overnight incubation (at least 16 h) at 4 °C, slides were washed in Tris/PBS buffer, respectively, and incubated for 1 h with AlexaFluor 488-conjugated goat anti-mouse/mouse anti-rabbit secondary antibodies, respectively (Cell Signaling Technology, Beverly, MA, USA; 1:500), washed, and embedded in PBS/glycerol mixture (9:1) with 3% DABCO.

During unperturbed replication (Figure 2A), forks are fired and progress according to the cell internal schedule [62,63]. Replication is a highly regulated process, involving early- and late-replicating DNA sites (Figure 2C), excess origin licensing [64,65], grouping of origins within replication factories [66], and the proper timing of everything to assure a flawless process of DNA synthesis in the shortest time possible [67]. From all the licensed origin sites, only a small percentage activate at once and recruit replisome proteins; the other origins remain dormant until they are replicated passively by an active fork or are required to activate when the closest fork stalls or collapses [66,68,69,70]. The mechanism by which dormant origins are kept inactive was elusive until recently. New studies show that even during proper replication, cells maintain low levels of ATR and checkpoint kinase 1 (Chk1) proteins (Figure 2A). The ATR/Chk1 signaling pathway usually mediates DNA repair and stabilizes stalled or collapsed replication forks [20,22,71]. However, ATR kinase activity also inhibits initiation of new origins in the absence of DNA damage if there are already active forks within the factory (Figure 2A and Figure 3C). The same pathway also seems to play a role in the inhibition of the initiation of replication factories that are scheduled to replicate later, after the currently active factory finishes DNA synthesis (Figure 2C).

In fact, hydroxyurea does not directly cause replication stress itself but rather does so indirectly via a depleted dNTP pool or through other byproducts of its activity [72,73], which depend on the species or the environment within the cell itself. Regardless, with RNR inactive, the free dNTPs are quickly depleted, especially during the S-phase (Figure 2), and replication activity is lowered (Figure 3A). Impaired DNA synthesis causes active forks to pause due to the absence of DNA synthesis substrates (Figure 2B and Figure 3A). The fork-stalling event is not abnormal in the cell—forks regularly stall at the genomic regions that are hard to replicate ([74]; e.g., those containing natural pausing elements, such as active transcription sites, centromeres, telomeres, and inactive origins) or at DNA lesions. So, the initial fork stall triggers a standard fork stabilization cell response that prevents the fork from collapsing [75]. Stalled forks then activate the S-phase checkpoint, which is dependent on ATR/Chk1 kinase activity (Figure 2B) [15,21,76,77,78]. The checkpoint is highly conserved and is a starting point for: (i) arresting cell cycle progression and delaying mitosis, (ii) stabilizing stalled forks, (iii) restoring RNR activity by promoting the synthesis of new β2 subunits, and (iv) activating neighboring dormant origins to carry on replication [21]. An important note here is that naturally occurring fork stalling is usually a local event affecting a small area of the DNA [79] during normal replication conditions, as there is a low density of natural pausing elements, whereas hydroxyurea affects the whole fork population.

The most fundamental function of S-phase checkpoint activation is a delay in entry into mitosis [15,80,81]. In mammals, the kinases involved in the pathway work in a hierarchical way, beginning with ATR, which detects replication protein A (RPA)-coated DNA replication-associated lesions. The signal is down-streamed through effector kinase Chk1 and then further to Cdc25 and Cdk1/cyclin B complexes, which finally arrest the cell in the S-phase until the initial problem is solved. Stalled forks cause the accumulation of ssDNA (Figure 3D) which quickly becomes coated by RPA proteins. Studies performed using Xenopus egg extract indicate that replication continues at stalled replication forks with synthesis and elongation of new primer sequences. This activity has been shown to also enhance Chk1 phosphorylation [82]. Additionally, the uncoupling of minichromosome maintenance (MCM) and DNA helicase has also been reported to contribute to the activation of the ATR-dependent checkpoint [83]. The sensors recruited to the stalled forks or damaged DNA site are not enough for the activation of ATR; rather, it is their interaction with specific activators that finally activates the checkpoint [21,84].

An increasing number of studies show that replication rapidly recovers after HU exposure, and that it is also connected to other S-phase checkpoint functions [73]. In budding yeast, suppressor of Mec1 lethality 1 (Sml1) normally suppresses the transcription of new RNR subunits. A Mec1–Rad53–Dun1 pathway activated by stalled forks (or simply at the beginning of the S phase) phosphorylates Sml1 and targets it for degradation [85,86]. Additionally, constitutive RNR transcription regulator 1 (Crt1) is also suppressed [87,88]. These events promote the synthesis of new RNR subunits and help in restoring the depleted dNTP pools. The results of research performed in mammalian cells reveal a similar RNR up-regulation pathway mediated by ATR–Chk1 kinases. Here, they help to accumulate ribonucleoside-diphosphate reductase subunit M 2 (RRM2), a subunit of RNR [28,89,90]. High levels of RRM2 have been proved to be efficient in suppressing the effects of ATR malfunctions [91].

Another major task of the S-phase checkpoint is the prevention of late origin firing and the stabilization of stalled replication forks (Figure 3B,C). In response to stalled forks, the ssDNA that becomes exposed is coated by RPA heterodimeric complexes which protect it and act as a landing platform to recruit ATR kinases [22,71,92]. This stalled fork maintenance event is linked to the ATR/Chk1 pathway of inhibition of origin firing. Also, other proteins like cohesins have also been reported to play a role in maintaining fork stability by promoting their restart [81,93,94,95,96]. In the presence of low dNTP pools, new origin initiation can lead to accumulation of ssDNA that can further lead to DNA breaks and overall large-scale DNA damage. In mammalian cells, inhibition of de novo origin firing is achieved by exhaustion of the RPA proteins coating the ssDNA at the sites of stalled replication forks [75,97].

## 4. The Consequences of S-Phase Checkpoint Malfunction

**Figure 4:**
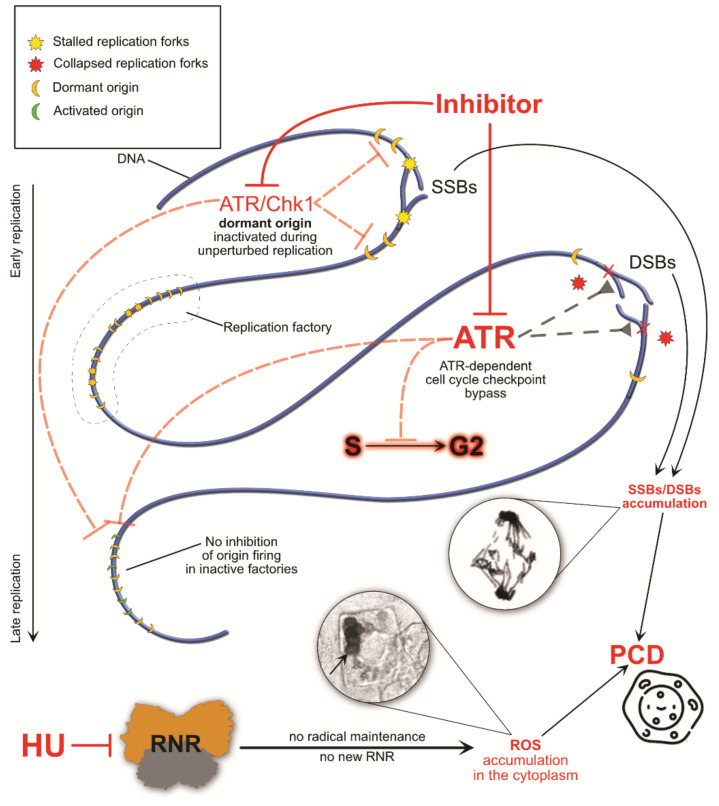
The consequences of ATR inhibition and malfunction of the S-phase checkpoint. A disrupted ATR/Chk1 pathway does not arrest the cell in the S phase and allows cycle progression with under-replicated DNA. This causes the accumulation of SSBs and DSBs, fork collapse, and deregulation of the inhibition of late origin firing. Moreover, a lack of radical maintenance and inhibition of new RNR synthesis leads to the accumulation of HU-induced ROS. The circled images show anaphase aberrations detected using Feulgen staining (**top**) and the accumulation of ROS (H_2_O_2_) in the cytoplasm in DAB-stained root meristem cells [99] (**bottom**) of *Vicia faba* subsp. *minor*. The experimental procedure for 3,3-diaminobenzidine (DAB) staining (to detect H_2_O_2_ by means of DAB polymerization) was as follows: roots of *V. faba* were submerged in Tris-buffered (10 mM Tris, 10 mM EDTA-2Na, 100 mM NaCl) DAB-HCl (1 mg mL^−1^; pH 7.5) dissolved in distilled water or in 2.5 mM HU (HU-treated plants). Following 24 h incubation, excised 3 mm root tips were fixed for 45 min in PBS-buffered 4% paraformaldehyde (20 °C), washed (3 times) with PBS, and placed in citric acid-buffered 2.5% pectinase (pH 5.0; 37 °C for 45 min). Digested root meristem cells were washed with PBS and squashed onto microscope slides in a mixture of glycerol and PBS (9:1; *v*/*v*). Production of H_2_O_2_ was observed under the microscope as reddish-brown areas in the cells (indicated by a black arrow in the circled image).

**Figure 5:**
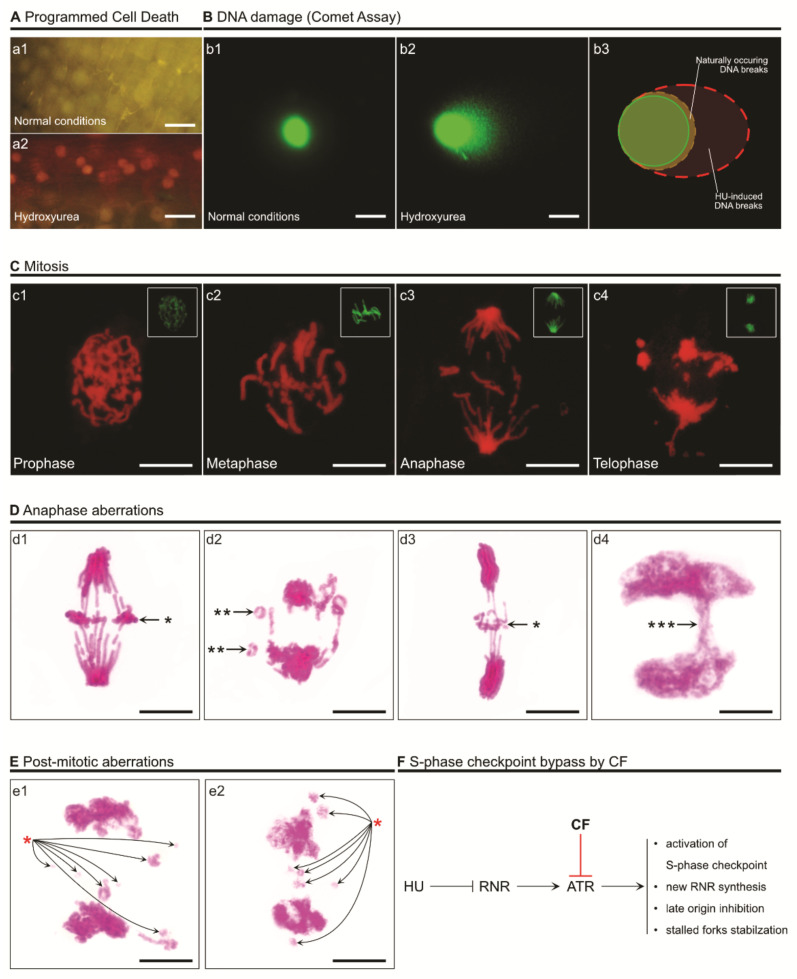
Visual examples of cell death, DNA damage, and chromosome breakage induced by overexposure to HU or bypassing of the S-phase cycle induced by 2.5 mM HU and 5 mM caffeine (CF) in root meristem cells of *Vicia faba* subsp. *minor*. (**A**) Programmed cell death (PCD) visible after double staining with acridine orange (AO) and ethidium bromide (EB) (**a1**,**a2**) [100]. AO is able to enter living cells and makes them appear green (**a1**). EB is taken up only when cell membrane integrity is compromised (usually in dead or dying cells), making them look red (**a2**). The range of colors (from green through yellow to red) indicates the existence of living, dying, and dead cells. The scale bar equals 50 µm. (**B**) DNA damage observed under normal conditions (**b1**) and after HU treatment (**b2**). The longer and more visible the comet’s tail is, the more DNA damage is present. DNA was stained with YOYO-1 (green; **b1**–**b3**). The scale bar equals 10 µm. (**C**) Feulgen-stained DNA and chromosome aberrations during mitosis, visible under a red spectrum in a fluorescent microscope (**c1**–**c4**) [101]. The experimental procedure for Feulgen staining (**d1**–**d4**,**e1**,**e2**) was as follows: root tips were fixed in cold absolute ethanol and glacial acetic acid (3:1, *v*/*v*) for 1 h, washed several times with ethanol, rehydrated, hydrolyzed in 4 M HCl (1.5 h), and stained with Schiff’s reagent (pararosaniline; Sigma-Aldrich, Saint Quentin, France) according to standard methods. After rising in SO_2_–water (three times) and distilled water, 1.5 mm long apical segments of roots were cut off, placed in a drop of 45% acetic acid, and squashed onto microscope slides. Following freezing using dry ice, coverslips were removed, and the dehydrated slides were mounted in Canada balsam. The top right inserts show mitotic morphology under normal conditions; DNA was also Feulgen-stained and the fluorescent image colors were edited to green for better visibility. The scale bars equal 10 µm. (**D**) Additional examples of anaphase aberrations (Feulgen staining under visible light). The single black asterisks (*) indicate mitotic chromosome fragments that were lost due to a lack of a connection to kinetochores (**d1**,**d3**); the double black asterisks (**) indicate O-shaped chromosomes (**d2**); the three asterisks (***) indicate a chromosome bridge (**d4**). The scale bars equal 10 µm. (**E**) Post-mitotic aberrations of decondensing DNA (Feulgen staining under visible light). Chromosome fragments (lost between opposite poles of the cell), i.e., later micronuclei, are indicated with a red asterisk (**e1**,**e2**). The scale bars equal 10 µm. (**F**) Short schematic of an S-phase checkpoint malfunction caused by caffeine (CF), as used in the presented experiments.

Prolonged HU exposure, a higher dosage, and/or an ineffective S-phase checkpoint may compromise the integrity of the DNA [75]. Numerous studies show that the absence of the S-phase checkpoint causes chromosome fragility [98]. The consequences are usually observed in mitosis, where condensed chromosomes are visible (Figure 4 and Figure 5). 

The fragile sites are most evident at “slow zones”, which are mostly late-replicating heterochromatin DNA sites [91,98,102,103]. The fragmentation of chromosomes is usually linked to low levels of RNR activity and decreased dNTP pools, to the point where fork integrity is compromised, leading to fork collapse and the formation of double DNA strand breaks (DSBs). Cells depleted of ATR or Mec1 are extremely fragile under exposure to HU, and artificially increased RNR activity usually alleviates some of this fragility [103]. Also, Rad 53 and Chk1 have been shown to up-regulate the local concentrations of dNTPs at active forks [10,104]. Another documented function of Chk1 is the prevention of enzymatic activity, leading to the cleaving of stalled replication forks [21,104]. In the absence of Chk1, fork stability is highly endangered and prone to collapse. It has been suggested that cells with an aberrantly functioning S-phase checkpoint express a different architecture of replication forks, such as reversals [21,105,106,107]. The results, however, seem to be contradictory. For instance, Rad52 is supposed to accumulate on the DNA in the S phase independently of replication activity [108]. Another study shows that higher Rad52 levels may depend on increased levels of ssDNA during replication [109]. It remains unclear whether the altered fork architecture in checkpoint depleted cells is pathological or normal. Fork reversal may be an intermediate step of stabilization and restart following stress [106,110,111]. In this scenario, a Rad51-dependent mechanism may turn stalled forks into reversed forks. In fact, in HU-exposed S-phase mutants, fork reversal is frequent [112]. Fork remodeling is related to its resection. Human translocase SWI/SNF-related matrix-associated actin-dependent regulator of chromatin subfamily A-like protein 1 (SMARCAL1) has been reported to be phosphorylated by ATR to induce fork remodeling of stalled forks, and ATR depleted cells express suppressed fragmentation of chromosomes with SMARCAL1 ablated [112,113]. Notably, a large portion of chromosome breakages stem from the activity of endonucleases (e.g., human Mus81) and factors expressing endonuclease activity (e.g., SLX4 or CtIP). These are mostly responsible for chromosome fragmentation in ATR-depleted cells [113,114].

Fork reversal is usually connected with higher levels of lagging and leading strand uncoupling in Rad53 mutants. Notably, exposure to HU leads to unloading of the PCNA protein (Figure 3B), mostly from the lagging strand [81,108,115]. It seems to be facilitated by S-phase checkpoint proteins, which supports the idea that, following HU-induced fork stalling, elongation of the lagging strand is inhibited [116,117]. The uncoupling of MCM and the DNA polymerases has also been proved to escalate ATR response [83]. MCM uncoupling usually generates ssDNA at the areas of fork junctions. Uncoupling may be prevented by pausing the complexes that maintain the coupling of MCM and the rest of the replisome [21]. This coordination seems to be deregulated when the checkpoint malfunctions. A failure in synchronization of the helicase movement may alter fork movement—possibly reversing the fork altogether [118].

When taken into consideration, a malfunction of the ATR-dependent S-phase checkpoint can lead to a variety of dysfunctions, such as fork stalling, unscheduled remodeling, and desynchronization of various proteins. The regulatory substrates that should be phosphorylated in order to maintain fork and DNA stability are also compromised. HU usually induces rapid phosphorylation of RPA2—a subunit of the ssDNA-detecting RPA protein. This phosphorylation is ATR dependent and is critical to secure DNA synthesis during replication stress [82,84,119]. HU activity also leads to the hyper-phosphorylation of the exonuclease 1 (Exo1) needed for resection of stalled or reversed forks that have been irreversibly altered [24,120,121]. In humans, Exo1 is phosphorylated in an ATR-dependent manner as well [122,123]. This endonuclease is essential in the process of tempering the 5′ flaps that tend to generate at uncoupled forks and in preventing the generation of reversed forks. ATR also mediates the phosphorylation of the MCM2 subunit of MCM in response to replication stress, which triggers the smaller intra-S checkpoint [76,83,124]. Other replisome components tend to be phosphorylated in this way as well, such as Psf1, a subunit of the GINS complex, or BLM helicase, which senses the ssDNA coated with RPA and is required for proper relocalization of Rad50/Mre11 proteins [69,70,125,126,127]. BLM is also needed for the suppression of dormant origin firing under replication stress [126]. 

Hydroxyurea has been reported to demonstrate rapid cell-killing abilities in various studies [31]. This phenomenon has been linked to the accumulation of ROS, which extensively oxidate macromolecules and greatly contribute to killing the cell (Figure 4). Prolonged HU exposure leads to cascade events related to membrane stress, which in turn produces hydroxyl radicals [128]. Increased iron levels may also contribute to the accumulation of ROS via Fenton chemistry [129]. HU exposure activates various pathways involved in radical maintenance. For instance, in *S. cerevisiae*, Yap (involved in redox homeostasis by activation of thioredoxin and thioredoxin reductase) and Aft (involved in iron homeostasis and DNA damage response by association with the TEAD transcription factors family) regulons are activated [16,130,131]. HU-generated ROS can also change the behavior of proteins containing Fe-S centers [16]. In *E. coli*, the YfaE enzyme containing an iron–sulfur cluster is an agent required for the maintenance of the diferric radical cofactor. Mutants depleted of YfaE have demonstrated higher sensitivity to HU due to the lack of radical regulation [43,132]. Without proper radical maintenance (in RNR or any other radical-based enzyme), radicals have a chance of leaking into the cytoplasm and easily generate superoxide [133]. In fact, many of the replication-related enzymes (e.g., primase, B-type DNA polymerases [134], DNA helicase XPD [135]) contain Fe-S clusters [136,137]. So, oxidative stress may also be the direct cause of replication inhibition, regardless of dNTP pool levels. This theory is consistent with research showing that, even without the depletion of dNTPs, replication is arrested [16].

## 5. Conclusions and Perspectives

**Figure 6:**
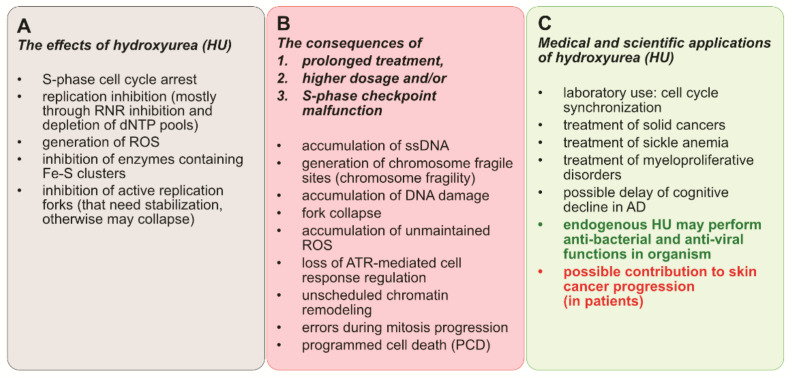
A summary of (**A**) the effects of HU exposure; (**B**) the consequences of high dosage, long treatment, and checkpoint deregulation; and (**C**) the medical and scientific applications of hydroxyurea.

One strength of our study was the in-depth analysis of the mechanism of action of HU. Even though hydroxyurea is a relatively small molecule, its impact on cell wellbeing may be significant (Figure 6). 

The mechanisms by which HU acts are usually indirect and mainly involve the induction of replication stress caused by fork stalling due to the depletion of dNTP pools, and the induction of ROS, which then contributes to cell death. The cytotoxic effects of HU, though very diverse and usually fatal, are mostly prominent in cells that lack the ATR-dependent S-phase checkpoint. Due to this fact, HU is a common anti-cancer drug. It does not significantly increase the level of DNA damage in patients with sickle cell disease [32], and recently it has been reported as a promising agent in preventing cognitive decline in Alzheimer’s disease. HU also increases fetal hemoglobin levels, which alters the kinetics of hemoglobin S polymerization [138], and has been shown to decrease the adhesion of blood cells to vascular endothelial cells [139]. Moreover, endogenous HU has been found in animals [60] and humans [140,141]. Even though its function remains unknown, the concentrations in certain tissues are high enough that it is actually effective against infections (either bacterial or viral). On the dark side of the matter, recent studies show new evidence of a possible contribution of HU to skin cancer progression [142], and the already common HU cancer treatments are still far from perfect. HU cell-killing mechanisms appear to be more general than previously thought, such as the wide range of Fe-S enzymes affected apart from RNR and the disruptions in the maintenance of free radicals throughout the cell. Hydroxyurea’s low stability in water and its degradation into byproducts that have different properties is also not favorable. However, hydroxyurea has also been shown to be the first-line drug in the context of myeloproliferative neoplasms (MPNs) and to play a role in modulating transcription through transcription factor activity and DNA methylation [143]. Our findings highlight a potential approach to amplifying replication stress-associated cytotoxicity of HU (e.g., through synthetic lethality).

## Abbreviations

The following abbreviations are used in this manuscript:

AD	Alzheimer’s disease
AO	Acridine orange
ATR	Ataxia telangiectasia and Rad3-related
BLM	Bloom syndrome protein
CF	Caffeine
Chk1	Checkpoint kinase 1
dNTP	Deoxyribonucleoside triphosphate
DSB	Double DNA strand break
EB	Ethidium bromide
Exo1	Exonuclease 1
HU	Hydroxyurea
MCM	Minichromosome maintenance
PCET	Proton-coupled electron transfer
PCNA	Proliferating cell nuclear antigen
RIFT-IR	Reaction-induced FT-IR
RNR	Ribonucleotide reductase
ROS	Reactive oxygen species
RPA	Replication protein A
RT	Radical transfer
